# Ankyloglossia unveiled: a clinical image highlighting the impact of tongue tie on oral mobility

**DOI:** 10.11604/pamj.2025.51.63.46390

**Published:** 2025-07-03

**Authors:** Meenakshi Dagar, Sheetal Asutkar

**Affiliations:** 1 Department of Shalyatantra, Mahatma Gandhi Ayurved College Hospital and Research Centre, Salod (H), Datta Meghe Institute of Higher Education and Research, Wardha, Maharashtra, India

**Keywords:** Ankyloglossia, frenotomy, speech therapy

## Image in medicine

A 12-year-old male patient presented with complaints of being unable to speak properly since birth. Clinical examination showed the frenulum attaches at the tip of the tongue, limiting its ability to extend outward, restricted movement of the tongue, and the patient was not able to extend his tongue beyond his lower lip. His medical and family history was clear; his parents told him he also had difficulty breastfeeding during his breastfeeding days. On systemic examination, there was no sign of hypotonia, cerebral palsy, which may be responsible for restricted tongue movement. Differential diagnoses included macroglossia, submucosal cleft palate, Down syndrome, Pierre Robin Sequence, and Ehlers-Danlos Syndrome (EDS). Diagnosis was based on clinical presentation and exclusion of other conditions. Treatment involved surgical intervention, frenotomy, and then advice for speech therapy and maintaining oral hygiene.

**Figure 1:**
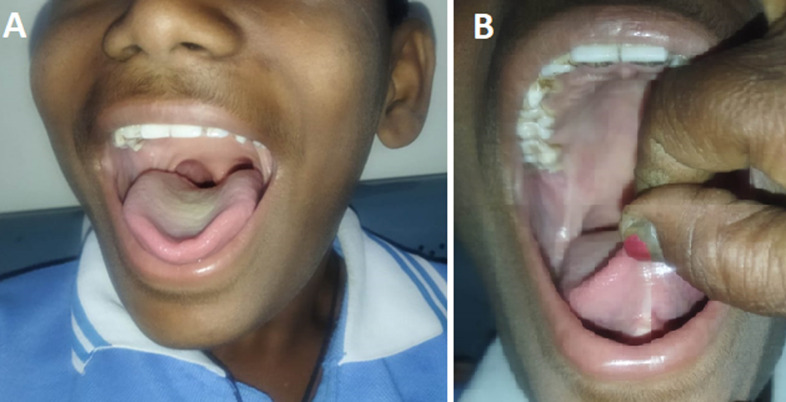
A) restricted movement of the tongue, not able to extend his tongue beyond his lower lip; B) frenulum attaches at the tip of the tongue

